# EDTA-assisted phase conversion synthesis of (Gd_0.95_RE_0.05_)PO_4_ nanowires (RE = Eu, Tb) and investigation of photoluminescence

**DOI:** 10.1080/14686996.2017.1338495

**Published:** 2017-06-28

**Authors:** Zhihao Wang, Ji-Guang Li, Qi Zhu, Zhengrong Ai, Xiaodong Li, Xudong Sun, Byung-Nam Kim, Yoshio Sakka

**Affiliations:** ^a^ Key Laboratory for Anisotropy and Texture of Materials, Northeastern University, Shenyang, China; ^b^ Institute for Ceramics and Powder Metallurgy, School of Materials Science and Engineering, Northeastern University, Shenyang, China; ^c^ Research Center for Functional Materials, National Institute for Materials Science, Tsukuba, Japan; ^d^ School of Environmental and Chemical Engineering, Dalian University, Dalian, China

**Keywords:** Phase conversion, GdPO_4_, nanowires, photoluminescence, EDTA, 40 Optical, magnetic and electronic device materials, 105 Low-Dimension (1D/2D) materials, 505 Optical / Molecular spectroscopy

## Abstract

Hexagonal (Gd_0.95_RE_0.05_)PO_4_·*n*H_2_O nanowires ~300 nm in length and ~10 nm in diameter have been converted from (Gd_0.95_RE_0.05_)_2_(OH)_5_NO_3_·*n*H_2_O nanosheets (RE = Eu, Tb) in the presence of monoammonium phosphate (NH_4_H_2_PO_4_) and ethylene diamine tetraacetic acid (EDTA). They were characterized by X-ray diffraction, thermogravimetry, electron microscopy, and Fourier transform infrared and photoluminescence spectroscopies. It is shown that EDTA played an essential role in the morphology development of the nanowires. The hydrothermal products obtained up to 180 °C are of a pure hexagonal phase, while monoclinic phosphate evolved as an impurity at 200 °C. The nanowires undergo hexagonal→monoclinic phase transformation upon calcination at ≥600 °C to yield a pure monoclinic phase at ~900 °C. The effects of calcination on morphology, excitation/emission, and fluorescence decay kinetics were investigated in detail with (Gd_0.95_Eu_0.05_)PO_4_ as example. The abnormally strong ^5^D_0_→^7^F_4_ electric dipole Eu^3+^ emission in the hexagonal phosphates was ascribed to site distortion. The process of energy migration was also discussed for the optically active Gd^3+^ and Eu^3+^/Tb^3+^ ions.

## Graphical abstract

**Figure:**
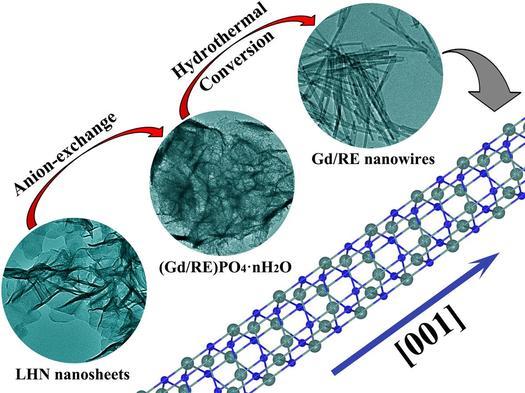

## 1. Introduction

One-dimensional (1D) nanostructures such as nanowires, nanorods, and nanotubes are drawing broad research interest owing to their unique physicochemical properties and applications in nanoscale devices, and a variety of strategies have been developed for their synthesis, typically including template-directed growth, the use of supersaturation control to modify the growth habit of the seed, and the use of capping reagents to kinetically control the growth rates of different crystal facets [1–5]. Rare-earth (RE) ions present numerous well-defined electronic transitions involving their 4f and 5d shells, and the RE-related materials have been applied in solid-state lasers, plasma display panels (PDPs), biolabeling, and so on [6–8]. Wang et al. [9] employed NaYF_4_:Yb,Er up-conversion nanoparticles as a novel fluorescent label for the detection of latent fingermarks with high sensitivity and broad applicability. As for 1D nanomaterials, Yang et al. [10] prepared nearly monodispersed and well-defined Gd_2_O_3_:Eu^3+^ nanorods and microrods through hydrothermal reaction followed by conventional annealing, and explored their size-dependent photoluminescence for potential application in high-performance phosphors. Ethylene diamine tetraacetic acid (EDTA) is well known to have strong ability to coordinate with metal ions with its four carboxyl groups (-COOH) and two nitrogen atoms, and has been tested to be useful in the morphology/structure modification and surface functionalization of an inorganic material [11]. Employing Na_2_EDTA as a capping reagent, Huang et al. [12] synthesized 1D nanobelts and nanorods of YVO_4_ via a facile hydrothermal route, and morphology-dependent luminescence of the Eu^3+^ doped YVO_4_ was also investigated.

One-dimensional rare-earth orthophosphates (REPO_4_) may find applications in phosphor displays and lighting, waveguide devices, fluorescence labels for biological detection, and solid-state lasers [13,14]. Gd^3+^ ion is well known to be strongly paramagnetic since it has the largest number (seven) of unpaired electrons in the lanthanide family, and is frequently used in the phosphor field as a sensitizer to improve excitation absorption and to enhance activator luminescence through energy transfer [15,16]. Therefore, gadolinium phosphate as a host lattice may combine the luminescence of the activator and the magnetic properties of Gd^3+^ [17]. Gadolinium phosphate has two basic structures depending on the extent of hydration, with the hydrous phase (GdPO_4_·*n*H_2_O) belonging to the rhabdophane type (hexagonal system, space group *P*3_1_21) and the anhydrous one (GdPO_4_) belonging to the monazite type (monoclinic, space group *P*2_1_/n). Huang et al. [18] developed a low-temperature solution approach to prepare hexagonal GdPO_4_·H_2_O nanorods with hydrogel-like property and proposed its utilization in encapsulation and drug release. Yu et al. [19] prepared monoclinic GdPO_4_:Eu^3+^ nanowires and nanorods via hydrothermal reaction and compared their luminescence properties. Through a melt-quenching method, Guo et al. [20] fabricated transparent glass ceramics containing GdPO_4_:Eu/Tb crystallites and manifested their possible application in lighting and luminescence fields. It is noteworthy that gadolinium phosphates were mostly investigated for the anhydrous monoclinic phase, since water of hydration always induces serious luminescence quenching.

Layered hydroxyl nitrate (LHN) compounds, exemplified by the group of RE_2_(OH)_5_NO_3_·*n*H_2_O, are attracting keen research interest during recent years owing to their unique layered structures and the rich electronic, optical, magnetic, and catalytic functionalities of the lanthanide (Ln) elements. We established in this work a new technique to generate (Gd_0.95_RE_0.05_)PO_4_·*n*H_2_O (RE = Eu, Tb) nanowires, employing nanosheets (thickness 3–5 nm) of the LHN as a sacrificial precursor, monoammonium phosphate (NH_4_H_2_PO_4_) as the phosphate source, and EDTA as a morphology modifier. It was also found that calcining the hydrated hexagonal nanowires at 500 °C did not bring obvious change to the morphology and phase purity but substantially enhanced their luminescence owing to dehydration. The effects of EDTA content, hydrothermal temperature on phase structure, morphology and photoluminescence of the nanowires were systematically studied, and the energy transfer from Gd^3+^ to Eu^3+^ and Tb^3+^ was also discussed.

## 2. Experimental details

### 2.1. Synthesis of LHN nanosheets and conversion into (Gd_0.95_RE_0.05_)PO_4_·nH_2_O (RE = Eu, Tb) nanowires

The starting gadolinium, europium, and terbium sources were Gd_2_O_3_, Eu_2_O_3_, and Tb_4_O_7_ (99.99% pure, Huizhou Ruier Rare-Chem. Hi Tech. Co. Ltd., Huizhou, China), and the nitrate solution of RE was prepared by dissolving the corresponding oxide with a proper amount of nitric acid. The reagents of ammonium water, EDTA (C_10_H_16_N_2_O_8_), and monoammonium phosphate (NH_4_H_2_PO_4_) were analytical grade products from Shenyang Chemical Reagent Factory (Shenyang, China).

The LHN precursor was produced via titrating nitrate solution of the rare-earth with ammonium water (1 mol/L) at ~4 °C [21]. For the phase conversion synthesis of phosphate, 0.5 mmol of LHN was dispersed in ~70 mL of deionized water, to which 1 mL of NH_4_H_2_PO_4_ solution (1 mol/L) was dropwise added, followed by magnetic stirring for 30 min and addition of a certain amount of EDTA. The resultant suspension was transferred into a Teflon-lined stainless steel autoclave of 100 mL capacity after magnetic mixing for 10 min, and the tightly sealed autoclave was then put into an electric oven preheated to a certain temperature for 24 h of reaction. After natural cooling to room temperature, the hydrothermal product was collected via centrifugation, washed with distilled water three times to remove byproducts, rinsed with absolute ethanol, and was finally dried in air at 70 °C for 24 h to yield a white powder for characterization and further processing. The effects of reaction parameters (Table 1) were studied with (Gd_0.95_Eu_0.05_)PO_4_ as example.

**Table 1.** A summary of experimental parameters.

Sample	Reaction time (h)	R = EDTA/(Gd_0.95_Eu_0.05_)	Reaction temperature (^o^C)
S1	0.5	0.5	Room temp.
S2	3	0.5	150
S3	6	0.5	150
S4	12	0.5	150
S5	24	0.5	150
S6	48	0.5	150
S7	24	0	150
S8	24	0.25	150
S9	24	0.75	150
S10	24	0.5	120
S11	24	0.5	180
S12	24	0.5	200

### 2.2. Characterization techniques

Crystal phases were identified by X-ray diffraction (XRD, Model PW3040/60, Philips, Eindhoven, The Netherlands, operated at 40 kV/40 mA), using nickel-ﬁltered Cu-*K*α radiation (λ = 0.15406 nm) and a scanning speed of 4.0^o^ 2θ per minute. Morphology and microstructure of the products were analyzed by field emission scanning electron microscopy (FE-SEM, Model JSM-7001F, JEOL, Tokyo, Japan) at an accelerating voltage of 15 kV and transmission electron microscopy (TEM, FEM-3000F, JEOL) at 200 kV. Fourier transform infrared spectroscopy (FTIR, Nicolet iS5, Thermal Fisher Scientific, New York, NY, USA) was combined with the standard KBr sample preparation method. Thermogravimetry (TG, Model TAS-200, Rigaku, Tokyo, Japan) of the powder was carried out in flowing oxygen gas with a heating rate of 10 °C/min. Photoluminescence spectroscopy and fluorescence decay kinetics of the phosphors were analyzed at room temperature with an FP-8600 fluorospectrophotometer (JASCO, Tokyo, Japan).

## 3. Results and discussion

### 3.1. Characterization and formation mechanism of the nanowires

**Figure 1.**
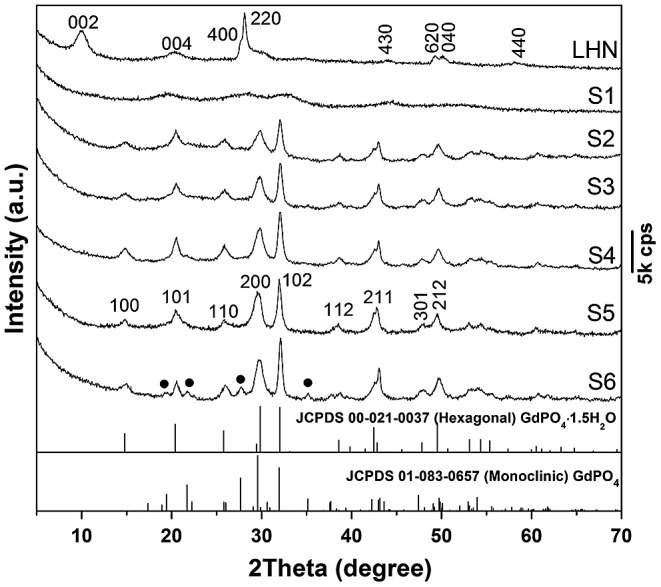
Powder XRD patterns for the original LHN and the products of S1 (anion exchange at room temperature for 30 min) and S2–S6 (hydrothermally reacted at 150 °C for 3, 6, 12, 24, and 48 h, respectively). The EDTA/(Gd_0.95_Eu_0.05_)^3+^ molar ratio (R) is 0.5 for S2–S6. The black dots in the XRD pattern of S9 denote monoclinic phosphate.

**Figure 2.**
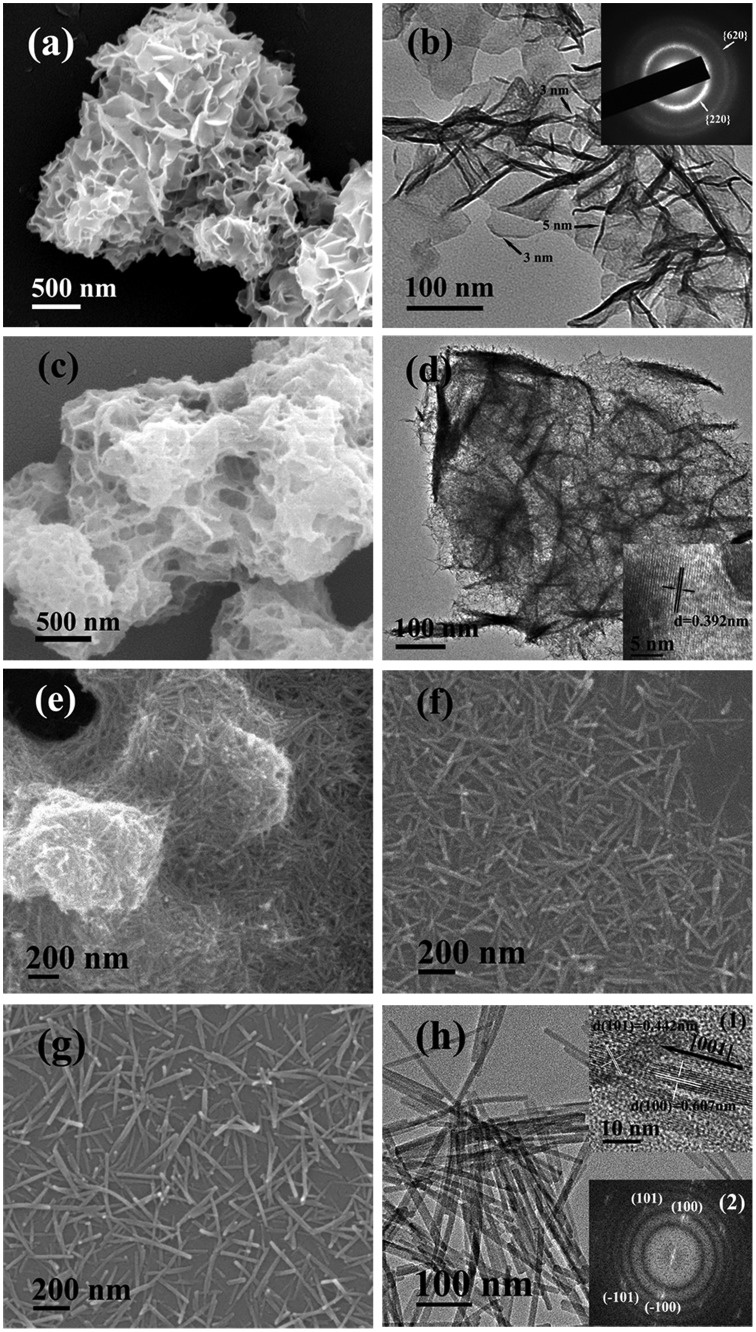
FE-SEM (a), (c), (e), (f), (g) and TEM (b), (d), (h) micrographs showing morphologies of the LHN (a), (b) and the four typical samples of S1 (c), (d), S2 (e), S3 (f), and S5 (g), (h). The insets in (b), (d), (h) are the corresponding SAED pattern, HR-TEM lattice image, and Fourier transform diffraction pattern, respectively. Samples S2, S3, and S5 were synthesized with an EDTA/(Gd_0.95_Eu_0.05_)^3+^ molar ratio (R) of 0.5.

Powder XRD analysis of the precursor (Figure 1) revealed a series of diffraction peaks that are characteristic of layered Ln_2_(OH)_5_NO_3_·*n*H_2_O (LHN) [21–25]. The crystal structure of LHN is built up via alternative stacking of the hydroxide main layers (*ab* plane) and exchangeable interlayer NO_3_^−^ along the *c*-axis. The occurrence of non-(00 *l*) diffractions thus indicates that the hydroxide layers are long-range ordered while the significantly broadened 00 *l* diffractions are owing to the thinness of the nanosheets, which are confirmed later by TEM analysis. The LHN diffractions are no longer observable for sample S1, implying that the hydroxide main layers have collapsed even after short (30 min) interaction with NH_4_H_2_PO_4_ at room temperature, as also seen from the results of morphology analysis (Figure 2). The broadened and diffuse diffraction peaks of S1 cannot be indexed to any crystalline form of rare-earth orthophosphate, due to the high water content and significantly nanocrystalline nature of the product [26]. Hexagonal GdPO_4_·1.5H_2_O (JCPDS File No. 00–021-0037, space group *P*3_1_21) crystallized in sample S2 via consumption of the unidentifiable phase of S1, despite a short hydrothermal reaction of 3 h. Better crystallinity (gradually sharper XRD diffractions) was achieved for the hexagonal phosphate by prolonging the hydrothermal reaction up to 12 h (S2–S4). Though the 24 h product (S5) was retained as pure hexagonal phase, the 48 h product (S6) crystallized with a minor amount of monoclinic phosphate (JCPDS No. 01–083-0657, space group: *P*2_1_/n) as impurity. The phase transition can be interpreted in view of thermodynamics, that is, the less stable hexagonal phase possesses higher free energy, which can be released to generate the stable monoclinic phase during prolonged hydrothermal reaction [27].

SEM and TEM images of the original LHN are presented in Figure 2(a) and (b), respectively. Micro-sized assemblies composed of LHN nanosheets (sputtered with gold for electrical conductivity) were clearly seen in Figure 2(a), while thicknesses of ~4 nm were observed for the primary nanosheets in Figure 2(b). Selected area electron diffraction (SAED, the inset in Figure 2(b)) clearly revealed the {220} and {620} planes, confirming that the hydroxide layers are well ordered as found via XRD. Interaction of LHN with the phosphate species dissociated from NH_4_H_2_PO_4_ at room temperature (sample S1) tends to disintegrate the hydroxide main layers into small particles (Figure 2(d)), though skeletons of the micron-sized assemblies were largely retained (Figure 2(c)). High-resolution TEM (HR-TEM) analysis (inset in Figure 2(d)) revealed that S1 has a crystalline structure, as evidenced by the well resolved lattice fringes with an inter-planar spacing of ~0.392 nm. Short-range crystallization (5–10 nm) was thus proposed to be the reason for the significantly broadened XRD peaks of S1 in Figure 1. Though the 3 h product (S2, Figure 2(e)) presents aggregates of needle-like particles, the 6 h product (S3, Figure 2(f)) and 24 h product (S5, Figure 2(g)) exclusively consist of monodispersed spicules. Longer reaction time favors Ostwald ripening and thus a more uniform particle morphology. Low-magnification TEM observation of S5 (Figure 2(h)) revealed that the particles have a length of about 300 nm and a width of about 10 nm. In view of the large aspect ratio (~30), the products can be ranked as nanowires. HR-TEM analysis (inset (1) in Figure 2(h)) indicates that each individual nanowire is well-crystallized with the (100) fringes running along the growth direction and spaced by 0.607 nm, and the (101) fringes can also be observed with a spacing of 0.442 nm. It can thus be figured out that the nanowire grows up along the [001] direction, as indicated by the arrow in the inset. Fourier transform of the lattice fringe yielded well-defined diffraction spots (inset (2) in Figure 2(h)), indicating a single crystalline nature of each nanowire. One conceivable advantage of this sacrificial precursor route is that the rare-earth cations are gradually released from the LHN nanosheets and also their skeletons (S1), which favors a more uniform morphology and better dispersion of the (Gd_0.95_Eu_0.05_)PO_4_·1.5H_2_O product.

**Figure 3.**
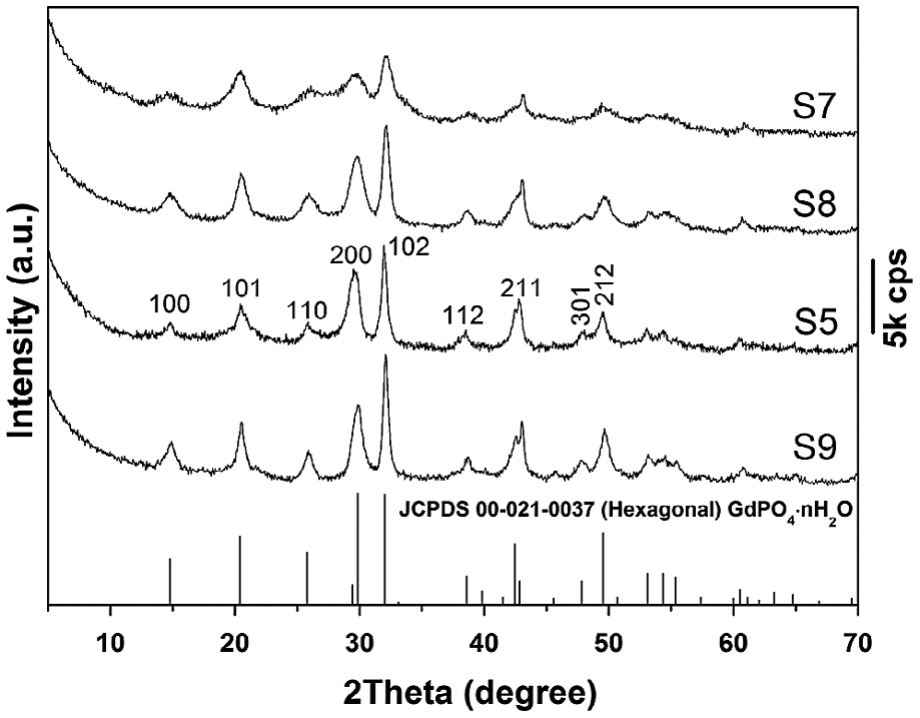
Powder XRD patterns for the products synthesized at the hydrothermal temperature of 150 °C and with EDTA/RE^3+^ molar ratio R of 0 (S7), 0.25 (S8), 0.5 (S5), and 0.75 (S9).

**Figure 4.**
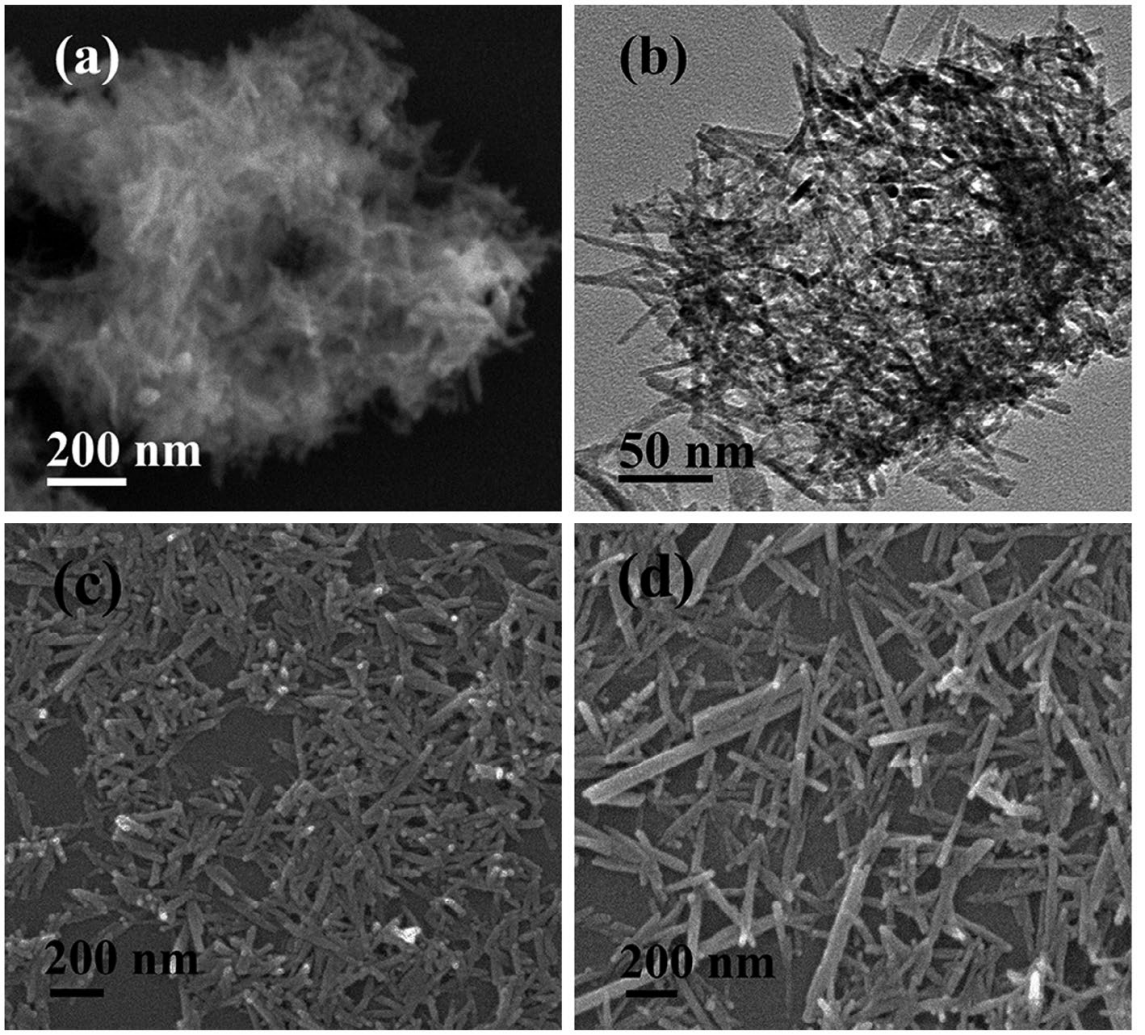
FE-SEM (a), (c), (d) and TEM (b) micrographs showing morphologies of the products obtained with EDTA/RE^3+^ molar ratio R of 0 (a, b; S7), 0.25 (c; S8), and 0.75 (d; S9).

The effects of EDTA on phase structure and morphology of the hydrothermal products were studied via altering the EDTA/RE^3+^ molar ratio R, and the results are shown in Figures 3 and 4, respectively. It is seen from Figure 3 that the EDTA-free product (S7) can be identified as hexagonal (Gd_0.95_Eu_0.05_)PO_4_·1.5H_2_O, though the diffraction peaks are broadened. Previous work showed that yttrium orthophosphate would crystallize in the tetragonal system (JCPDS No. 00–083-0658, space group: *I*4_1_/*amd*) under similar hydrothermal conditions [28], manifesting the decisive role of RE^3+^ size in phase selection. Increasing EDTA addition continuously improved crystallinity of the hydrothermal product, but did not alter phase purity (S8, S5, and S9, Figure 3). The significant effects of EDTA can also be perceived through comparing morphologies of the products shown in Figure 4, where it is seen that the sample synthesized without EDTA (Figure 4(a), (b)) consists of agglomerates entangled from underdeveloped acicular particles while that made with a small amount of EDTA (R = 0.25) is composed of much better dispersed nanowires/nanorods with an aspect ratio of ~10 (Figure 4(c)). Increasing the ratio R to 0.5 led to monodispersed nanowires (Figure 2(g)), while significantly longer rods with larger diameters (aspect ratio: ~20–30) appeared in the product at the even larger R value of 0.75 (Figure 4(d)).

The phase structure and crystal shape of a hydrothermal product are known to be synergistically determined by additives and crystallization habit [29]. Lanthanide orthophosphates possess extremely low water solubility (solubility product is on the order of 10^−25^ to 10^−27^) [30], and, accordingly, the S1 product would dissociate in an extremely low degree according to the following equation:



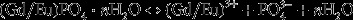



The addition of EDTA lowers the concentration of Gd^3+^/Eu^3+^ in the solution through chelation and thus right-shifts the equation, which speeds up the process of Ostwald ripening to yield the larger and better dispersed nanowires shown in Figures 4 and 2(g). Meanwhile, EDTA may cap crystal surfaces, lowering the energy cost for creating new surfaces and hence also encouraging the formation of relatively dispersed crystals [31]. It should be noted that the chelating ability of EDTA increases with increasing content (the R value), which enhances the dissolution of phosphate and lowers the nucleation rate. This is in accordance with our observation that increasing the R ratio from 0.25 to 0.75 yielded gradually larger particles and no solid particles could be obtained at the high R ratio of 1.

**Figure 5.**
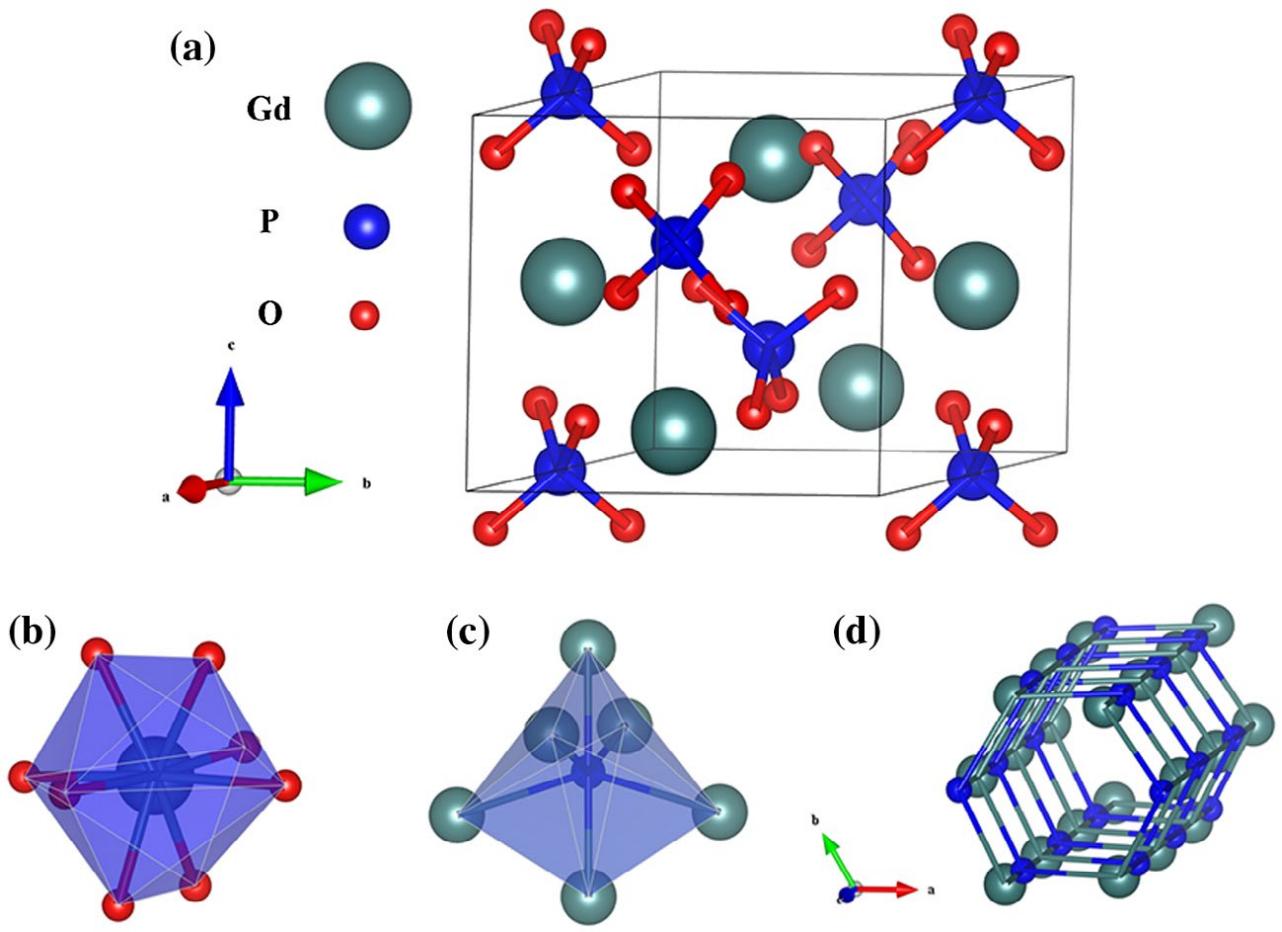
Schematic of the crystal structure of hexagonal GdPO_4_·*n*H_2_O (a), GdO_8_ dodecahedron (b), and P-Gd octahedron (c). (d) shows the open tunnels running through the structure along the *c* axis (oxygen atoms are omitted for clarity).

The hexagonal unit cell of GdPO_4_·*n*H_2_O (Figure 5(a), realized with the Vesta software [32]) consists of three formula units, where two sets of oxygen atoms, differing in Gd-O bond length, are coordinated to Gd^3+^ to form GdO_8_ dodecahedron. As a result, the Gd^3+^ ions are residing at the distorted *D*_2_ crystallographic sites (Figure 5(b)) [33]. Each phosphate group (PO_4_ tetrahedron) in the structure is coordinated to six Gd^3+^ to form P-Gd octahedron (Figure 5(c)), where the P atom assumes a *C*_2_ point symmetry [34]. The crystal structure contains -Gd^3+^-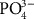-Gd^3+^- chains running along the *c* axis to form open tunnels, where hydration water is accommodated (Figure 5(d)). Murphy et al. previously reported that the activation energy for the growth of hexagonal LnPO_4_ along the *c*-axis is lower than that perpendicular to the *c*-axis [35], which may account for the crystallization of [001]-oriented (Gd,Eu)PO_4_·*n*H_2_O nanowires in this work (inset (1) in Figure 2(h)).

**Figure 6.**
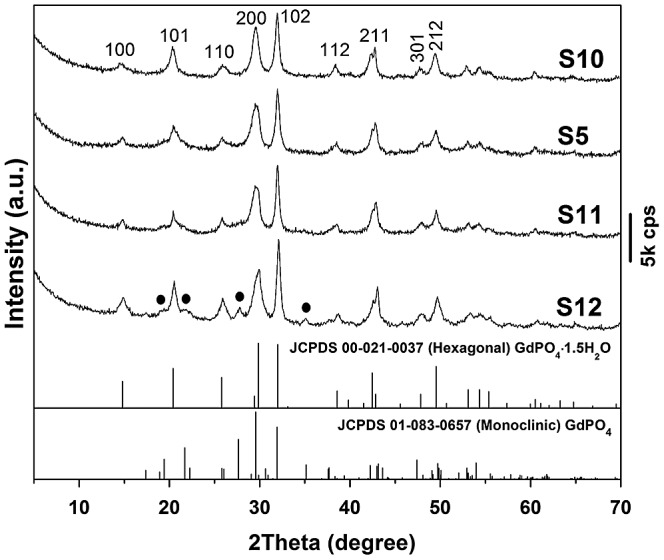
Powder XRD patterns for the products synthesized at the different hydrothermal temperatures of 120 °C (S10), 150 °C (S5), 180 °C (S11), and 200 °C (S12). The EDTA/(Gd_0.95_Eu_0.05_)^3+^ molar ratio (R) is 0.5 in each case. The black dots in the pattern of S12 denote monoclinic phosphate.

The effects of hydrothermal temperature on phase structure and morphology of the nanowires were investigated in Figure 6 and Figure S1, respectively. It was found from Figure 6 that the 120–180 °C products are all of the hexagonal phase while the even higher temperature of 200 °C induced partial crystallization of monoclinic phosphate (denoted with black dots). The reason is similar to that discussed for the effects of reaction time (Figure 1). Although SEM (Figure S1(a), (b)) and TEM (Figure S1(d), (e)) observations revealed that the nanowires are less uniform in length and width for samples S10 and S11, SAED (insets in Figure S1(d), (e)) indicated that each individual wire is of single crystalline. Through morphology comparison, it is clear that 150 °C is the optimal hydrothermal temperature to generate nearly monodispersed nanowires.

### 3.2. Thermal behaviors of the nanowires

**Figure 7.**
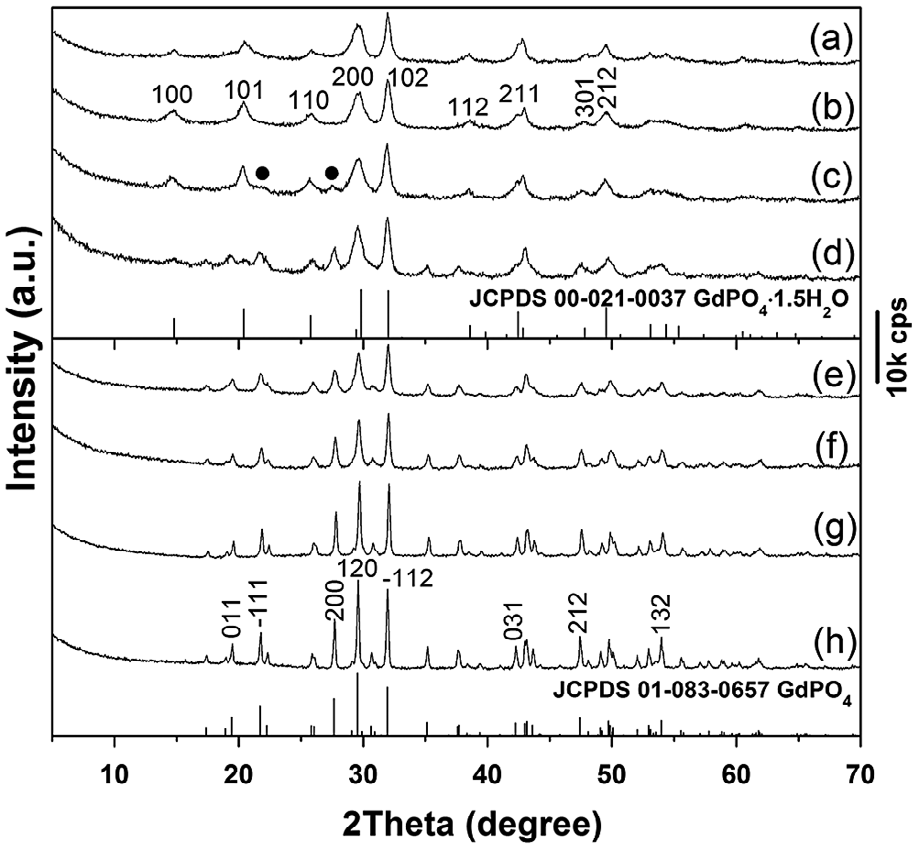
Powder XRD patterns for sample S5 (a) and the products calcined from S5 at: (b) 500, (c) 600, (d) 800, (e) 900, (f) 1000, (g) 1100, and (h) 1200 °C. The black dots in (c) denote monoclinic phosphate.

Thermal behaviors of the nanowires were investigated with sample S5 as example. Figure 7 compares XRD patterns of the original nanowires (Figure 7(a)) and those calcined under flowing O_2_ at various temperatures for 2 h. It is seen that the hexagonal crystal structure can be well retained up to 500 °C (Figure 7(b)) while calcination to 600 °C led to partial crystallization of the monoclinic phase (JCPDS No. 01–083-0657; space group: *P*2_1_/n; labeled in Figure 7(c) with black dots). TG analysis (Figure S2) showed that the sample completely dehydrates up to 570 °C via two major stages, with the first one (up to ~150 °C) due to evaporation of surface-adsorbed water, and the second one (150–570 °C) owing to the removal of hydration water. It can thus be inferred from Figure 7(c) and Figure S2 that complete dehydration tends to collapse the hexagonal structure or, in other words, the metastable hexagonal phase is stabilized by hydration water. The *n* value of the (Gd_0.95_Eu_0.05_)PO_4_·*n*H_2_O nanowires was determined to be around 1.0 from the total weight loss of 6.76%. Calcining to 900 °C caused full crystallization of the monoclinic phase (Figure 7(e)), and the higher temperature products (up to 1200 °C) remained as monoclinic phosphate. Improved crystallinity owing to crystal perfection and crystallite growth was observed with increasing temperature of calcination, as evidenced by the successively sharper and stronger XRD diffractions (Figure 7(f)–(h)). The monoclinic (Gd_0.95_Eu_0.05_)PO_4_ powders had average crystallite sizes of ~29, 37, 78, and 90 nm after calcination at 900, 1000, 1100, and 1200 °C, respectively, as estimated from the width of the (120) diffraction peak using the Scherrer formula.

**Figure 8.**
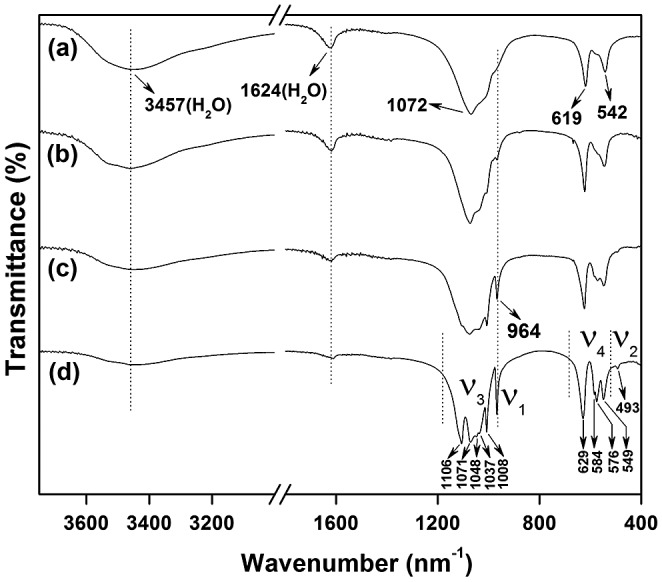
FTIR spectra for sample S5 (a) and the products calcined from S5 at 500 (b), 900 (c), and 1200 °C (d).

Figure 8 compares FTIR spectra of the nanowires before and after calcination, from which it can be seen that dehydration weakens the characteristic vibrations of water molecules (*ν*_1_ and *ν*_3_ at 3457 cm^−1^ and *ν*_2_ at 1624 cm^−1^). A characteristic profile of orthophosphate groups under *C*_2_ symmetry is observed in the IR spectra of the hexagonal phase (Figure 8(a), (b)). For *C*_2_ symmetry, each of the four absorption bands (*ν*_1_, *ν*_2_, *ν*_3_, and *ν*_4_) of 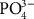 is theoretically active [34], as observed in this work for the P-O symmetric stretching (*ν*_1_, A) vibration as a weak band at ~964 cm^−1^, the P-O antisymmetric stretching (*ν*_3_, A + 2B) as an intense band centered at ~1072 cm^−1^, and the O-P-O antisymmetric deformation (*ν*_4_, A + 2B) bands in the 500–700 cm^−1^ region. The *ν*_2_ vibration is nonetheless too weak to be recorded, which is coinciding with the report of Hezel and Ross [34]. The hexagonal to monoclinic phase conversion changes the crystallographic sites of 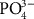 into the lower symmetry of *C*_1_, which allows better resolved vibrations [36], as can be seen from Figure 8(c, d). The vibration region of 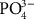 anions in the monoclinic (Gd_0.95_Eu_0.05_)PO_4_ calcined at 1200 °C presents *ν*_1_ vibration as a sharp band at ~964 cm^−1^, *ν*_2_ vibration as a shallow band at ~493 cm^−1^, *ν*_3_ stretching band as five observable components at ~1008, 1037, 1048, 1071, and 1106 cm^−1^, and *ν*_4_ vibration as several shallow absorptions with frequencies at ~549, 576, 584, and 629 cm^−1^. The above observations agree with the results of Kijkowska [36].

Figure S3 shows typical FE-SEM morphologies of the calcination products. Although calcination at 500 °C did not affect phase purity of the hexagonal (Gd_0.95_Eu_0.05_)PO_4_·*n*H_2_O compound, a slight collapse of the nanowires happened (Figure S3(a)). Calcining to 600 °C induced substantial destruction of the 1D morphology, mainly owing to dehydration. The original nanowire morphologies are hardly observable for the 900 and 1000 °C products because of the re-constructive type hexagonal→monoclinic phase transition. Abrupt particle coarsening took place at 1100 °C, which corresponds well to the results of crystallite analysis (37.6 and 77.5 nm at 1000 and 1100 °C, respectively).

The Tb^3+^ doped nanowires of (Gd_0.95_Tb_0.05_)PO_4_·*n*H_2_O were also synthesized under the conditions used for sample S5. XRD analysis (Figure S4) and FE-SEM observation (Figure S5) indicated that (Gd_0.95_Tb_0.05_)PO_4_·*n*H_2_O similarly crystallized as hexagonal nanowires, and phase purity and the 1D morphology were well retained after 500 °C calcination.

### 3.3. Optical properties of the (Gd_0.95_Eu_0.05_)PO_4_ and (Gd_0.95_Tb_0.05_)PO_4_ phosphate phosphors

**Figure 9.**
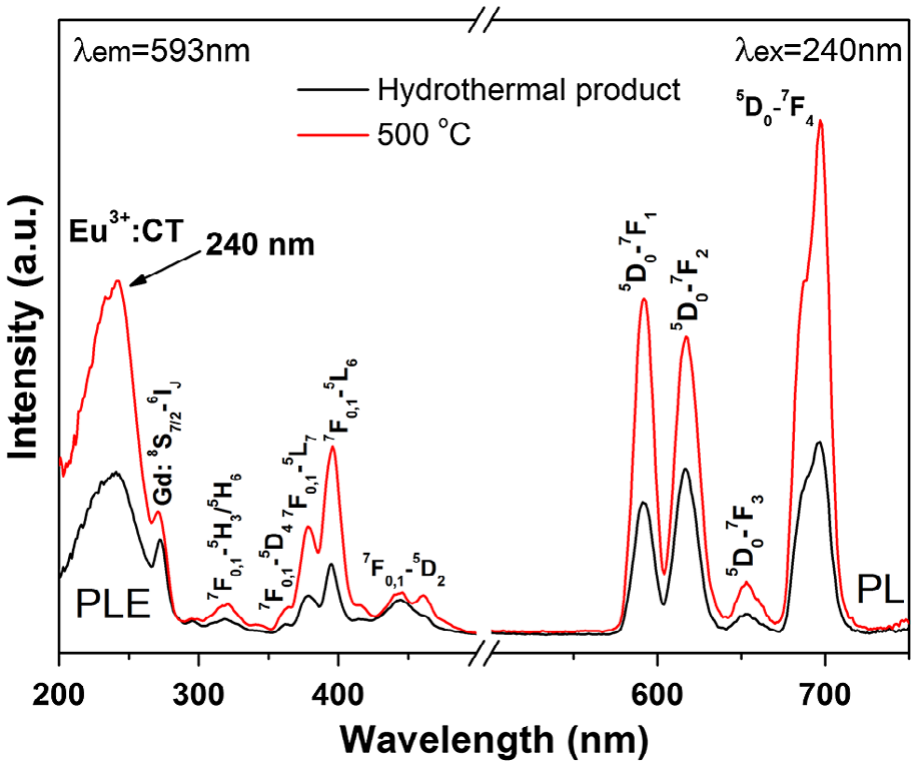
PLE and PL spectra of sample S5 and its product calcined at 500 °C. CT stands for charge transfer.

Since calcination at 600 °C induced partial crystallization of the monoclinic phase and substantial destruction of the 1D morphology of the nanowires, the Eu/Tb-doped hexagonal nanowires were annealed at 500 °C to investigate their luminescence properties. Figure 9 shows photoluminescence excitation (PLE) and photoluminescence (PL) spectra for the hexagonal (Gd_0.95_Eu_0.05_)PO_4_·*n*H_2_O and (Gd_0.95_Eu_0.05_)PO_4_. The band observed at ~240 nm in the PLE spectra corresponds to the excitation of electrons from the 2p orbital of O^2-^ in the PO_4_^3-^ group to the 4f orbital of Eu^3+^, which is known as charge transfer. The weak shoulder at ~272 nm is attributed to the ^8^S_7/2_→^6^I_*J*_ intra-4f^7^ transition of Gd^3+^. The intra-4f^6^ transitions of Eu^3+^ were observed at ~318 nm for the ^7^F_0,1_→^5^H_3_/^5^H_6_, 363 nm for the ^7^F_0,1_→^5^D_4_, 378 nm for the ^7^F_0,1_→^5^L_7_, 396 nm for the ^7^F_0,1_→^5^L_6_, and 448 nm for the ^7^F_0,1_→^5^D_2_ transitions, as labeled in the figure. Upon UV excitation at 240 nm, the two hexagonal phosphates exhibit emissions ranging from 500 to 750 nm, which are associated with transitions from the excited ^5^D_0_ state to the ^7^F_*J*_ (*J* = 1–4) ground states of Eu^3+^ as labeled in the figure, with the ^5^D_0_→^7^F_4_ transition (~697 nm) being the most prominent. Intensity of both the PLE and PL bands were greatly enhanced by calcination at 500 °C, owing to significant dehydration (Figure S2) and elimination of luminescence-quenching defects such as surface dangling bonds and particularly hydroxyls [5].

The relative intensities of transitions from the ^5^D_0_ excited state to the different *J* levels of the ground state depend on the site symmetry of Eu^3+^ and can be described in terms of the Judd-Ofelt theory, which predicts that the ^5^D_0_→^7^F_1,3_ magnetic dipole transition is permitted while the ^5^D_0_→^7^F_0,2,4_ electric dipole transition is forbidden, and the latter is allowed only on condition that the Eu^3+^ ions occupy a site without an inversion center [37,38]. As mentioned above, the Eu^3+^ ions are expected to replace Gd^3+^ to inherit the centrosymmetric *D*_2_ point symmetry in hexagonal (Gd_0.95_Eu_0.05_)PO_4_·*n*H_2_O and (Gd_0.95_Eu_0.05_)PO_4_. The abnormally strong ^5^D_0_→^7^F_2,4_ emissions observed for the above two phosphates (Figure 9) may be understood from the Ω_*λ*_ intensity parameter. It is known that the intensities of the ^5^D_0_→^7^F_2_ and ^5^D_0_→^7^F_4_ transitions are governed by the effective operators of Ω_2_U^(2)^ and Ω_4_U^(4)^, respectively, where U^(*λ*)^ is the unit tensor operator [39]. Nanomaterials generally show lattice distortions, unlike bulk crystals, and hence it is plausible to say that in this work the Eu^3+^ activators have a lower site symmetry distorted from *D*_2_. The distortion may make the ligand field-/polarizability-related parameters larger, and thus enlarges the Ω_2,4_ parameters to lead to the strong ^5^D_0_→^7^F_2_ and particularly ^5^D_0_→^7^F_4_ electric dipole transitions [40].

**Figure 10.**
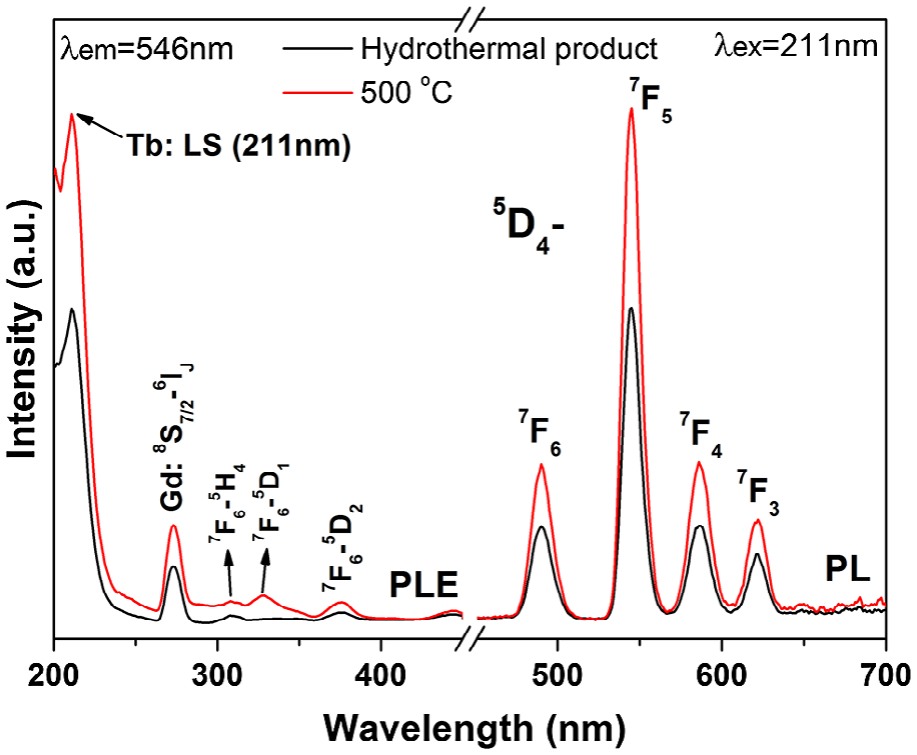
PLE and PL spectra of (Gd_0.95_Tb_0.05_)PO_4_·*n*H_2_O and its product calcined at 500 °C.

The excitation and emission behaviors of Tb^3+^ in the hexagonal phosphates of (Gd_0.95_Tb_0.05_)PO_4_·*n*H_2_O and (Gd_0.95_Tb_0.05_)PO_4_ are studied in Figure 10. The excitation spectra of the two samples recorded by monitoring the ^5^D_4_→^7^F_5_ green emission at 546 nm consist of two bands in the short UV region (up to 300 nm) for the low-spin inter-configurational 4f^8^→4f^7^5d^1^ transition of Tb^3+^ (LS, at ~211 nm) and the ^8^S_7/2_→^6^I_*J*_ intra-4f^7^ transition of Gd^3+^ (at ~272 nm) [23]. The other bands in the longer UV region of 300–400 nm arise from intra-4f^8^ excitation transitions of Tb^3+^, as labeled in the figure. The whole excitation spectrum is dominated by the LS transition at 211 nm. The PL spectra obtained under 211 nm excitation consist of emissions ranging from 450 to 700 nm, which are associated with transitions from the excited ^5^D_4_ state to the ^7^F_*J*_ (*J* = 3–6) ground states. The strongest emission is located at ~546 nm (^5^D_4_→^7^F_5_ transition), typical of a green color. The hexagonal (Gd_0.95_Eu_0.05_)PO_4_ and (Gd_0.95_Tb_0.05_)PO_4_ anhydrous nanowires, with enhanced luminescence and favorable dispersity, may find applications in the fields such as biolabeling, optoelectronics, and sensing [41].

**Figure 11.**
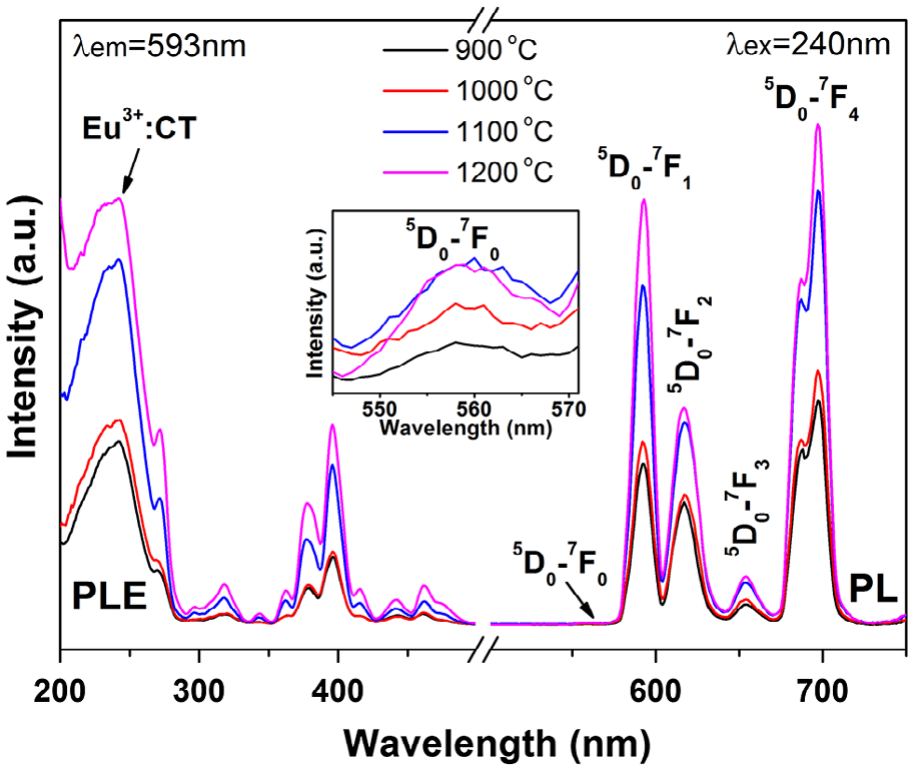
PLE and PL spectra of the monoclinic products calcined from sample S5 at the various temperatures indicated in the figure.

PLE/PL spectra of the calcination-derived (Gd_0.95_Eu_0.05_)PO_4_ monoclinic phosphates are presented in Figure 11. It is seen that both the excitation and emission intensities steadily increase with increasing temperature of calcination. The increment from 1000 to 1100 °C is the most prominent, which corresponds well to the greatly enhanced crystallization observed from Figure 7. In monoclinic GdPO_4_, Yaiphaba et al. reported that the Eu^3+^ ion is surrounded by nine oxygen atoms to form EuO_9_ with different Eu-O bond lengths, residing at the highly asymmetric *C*_1_ site [42]. This agrees with the presence of ^5^D_0_→^7^F_0_ transition (inset in Figure 11).

The energy transfer from Gd^3+^ to Eu^3+^ was analyzed for the 1000 °C sample through exciting the ^8^S_7/2_→^6^I_*J*_ transition of Gd^3+^ at 272 nm, and the resultant PL spectrum, which consists of the characteristic ^5^D_0_→^7^F_*J*_ (*J* = 1–4) emissions of Eu^3+^, is shown in Figure S6(a). The process of energy transfer involved in Eu^3+^ emission is schematically shown in Figure S6(b). That is, exciting the (Gd_0.95_Eu_0.05_)PO_4_ phosphor at 272 nm raises electrons from the ^8^S_7/2_ ground state to the ^6^I_*J*_ energy level of Gd^3+^, followed by relaxation to the ^6^P_7/2_ level. The ^6^P_7/2_ electrons first relax to the higher ^5^D_*J*_ (*J* = 1, 2, 3) excited states of Eu^3+^, followed by further relaxation to the lowest-lying ^5^D_0_ excited level in a rapid non-radiative way. Subsequently, f–f transitions of Eu^3+^ take place from the ^5^D_0_ state to the ^7^F_*J*_ (*J* = 0, 1, 2, 3, 4) ground states. Tb^3+^ emissions enabled by Gd^3+^→Tb^3+^ energy transfer were similarly achieved, as seen in Figure S7(a), (b).

**Figure 12.**
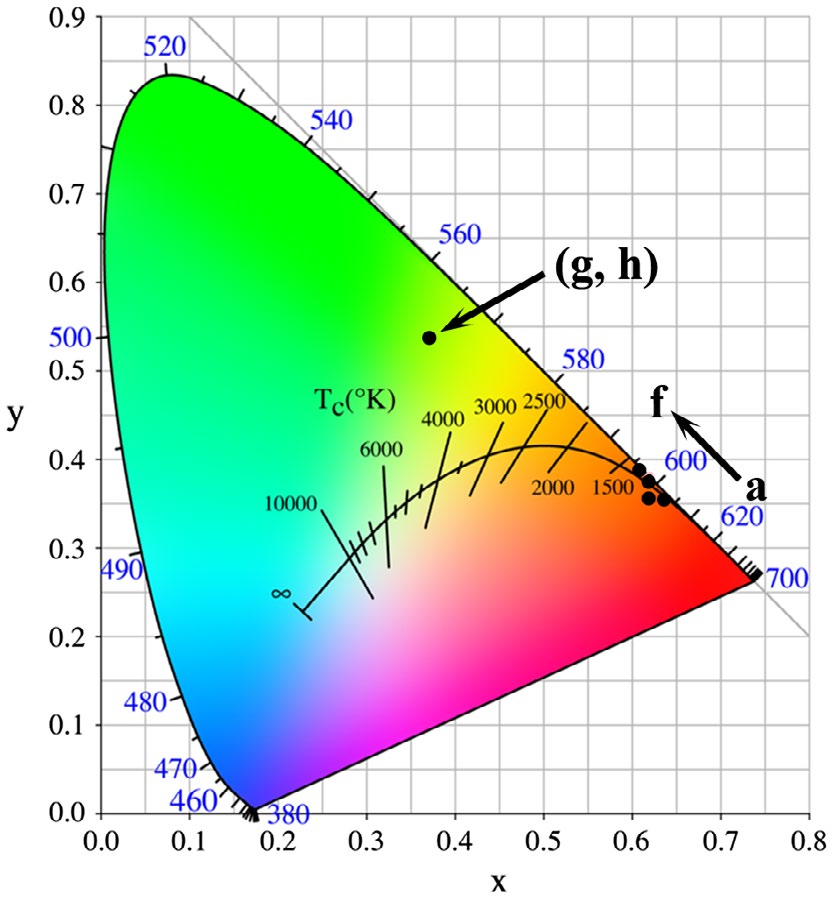
CIE chromaticity diagram for the emissions of Eu^3+^ (a)–(f) and Tb^3+^ (g), (h) in gadolinium phosphates.

Fluorescence decay kinetics of the 593 nm red emission of Eu^3+^ and the 546 nm emission of Tb^3+^ have been investigated under 240 nm (for Eu^3+^) and 211 nm (for Tb^3+^) excitations, and the results are presented in Figure S8(a) and (b), respectively. The decay curves can be well fitted with the single-exponential function of *I* = *A*exp (−*t*/*τ*) + *B*, where τ is the fluorescence lifetime, *t* the delay time, *I* the relative intensity, and *A* and *B* are constants. It is seen from Figure S8(a) that the lifetime of Eu^3+^ was increased continuously from ~3.66 to 6.70 ms by calcination up to 1100 °C and then decreased to ~4.66 ms after 1200 °C calcination. The constant increase is primarily owing to the removal of luminescence-quenching defects and surface species, while the subsequent decrease is mainly due to increased effective refractive index of the phosphor by significant crystallite/particle coarsening [43]. Slight lifetime elongation by calcination was also observed for the Tb^3+^ emission. The emission of Eu^3+^ under 240 nm excitation was calculated from Figures 9 and 11 to have Commission internationale de l’éclairage (CIE) chromaticity coordinates of around (0.63, 0.36) for the 150 °C hydrothermal and 500 °C calcination products, (0.62, 0.36) for the 900 °C, (0.62, 0.38) for the 1000 °C, and (0.61, 0.39) for the 1100 and 1200 °C calcination products (Figure 12(a)–(f)). The color coordinates slightly yet steadily shift to the orange color region for the sample calcined at a higher temperature (a–f, arrow direction), owing to faster intensity increase of the ^5^D_0_→^7^F_1_ orange-red emission than the ^5^D_0_→^7^F_2,4_ red emissions as inferred from the intensity ratio of these transitions (Figure S9). The two Tb^3+^ containing samples were calculated to have the same CIE chromaticity coordinates of about (0.37, 0.54), falling in the green area of the diagram (points g and h).

## 4. Conclusions

Hexagonal (Gd_0.95_RE_0.05_)PO_4_·*n*H_2_O (RE = Eu, Tb) nanowires have been hydrothermally converted from the nanosheets of layered rare-earth hydroxyl nitrate. The nanowires were determined to grow along the [001] crystallographic direction, and EDTA was found to play a decisive role in morphology evolution of the nanowires. Both the hexagonal structure and nanowire morphology can be retained after calcination at 500 °C, and monoclinic (Gd_0.95_Eu_0.05_)PO_4_ emerges as a pure phase at 900 °C. The Eu^3+^ and Tb^3+^ activators exhibit their characteristic ^5^D_0_→^7^F_*J*_ and ^5^D_4_→^7^F_*J*_ emissions under excitation with peak wavelengths of the O^2−^→Eu^3+^ charge transfer and low-spin 4f^8^→4f^7^5d^1^ transition bands, respectively. The abnormally strong ^5^D_0_→^7^F_4_ electric dipole Eu^3+^ emission in the hexagonal phosphates was ascribed to the site distortion. Exciting the Gd^3+^ ions in the host lattice with the ^8^S_7/2_→^6^I_*J*_ transition at ~272 nm also produces the characteristic emissions of Eu^3+^ and Tb^3+^, owing to Gd^3+^→Eu^3+^/Tb^3+^ energy transfer.

## Disclosure statement

No potential conflict of interest was reported by the authors.

## Funding

Z.H. Wang acknowledges financial support from the China Scholarship Council for his overseas PhD study [Contract No. 201606080030]. This work was supported in part by the National Natural Science Foundation of China [grant number 51672039], the Fund of the State Key Laboratory of Advanced Technologies for Comprehensive Utilization of Platinum Metals [SKL-SPM-201505], and the Fundamental Research Funds for the Central Universities [Grants No. N160206001; N160204008].

## Supplemental data

The supplemental material for this paper is available online at https://doi.org/10.1080/14686996.2017.1338495.

## Supplementary Material

Supplementary_information.doc

## References

[1] Wang ZL Characterizing the structure and properties of individual wire-like nanoentities. Adv Mater. 2000;12:1295–1298. 10.1002/(ISSN)1521-4095

[2] Xia YN, Yang PD, Sun YG, et al One-dimensional nanostructures: synthesis, characterization, and applications. Adv Mater. 2000;15:353–389.

[3] Wang X, Sun XM, Yu DP, et al Rare earth compound nanotubes. Adv Mater. 2003;15:1442–1445. 10.1002/(ISSN)1521-4095

[4] Wang X, Li YD Synthesis and characterization of Lanthanide Hydroxide single-crystal nanowires. Angew Chem Int Ed. 2002;41:4790–4793. 10.1002/(ISSN)1521-3773 12481359

[5] Wang ZH, Li J-G, Zhu Q, et al. Tartrate promoted hydrothermal growth of highly [001] oriented (La_0.95-_ ^*x*^Bi^*x*^Eu_0.05_)PO_4_ (*x*=0-0.01) nanowires with enhanced photoluminescence. Mater Des. 2017;126:115–122.

[6] Wang X, Li YD Rare-Earth-Compound nanowires, nanotubes, and fullerene-like Nanoparticles: synthesis, characterization, and properties. Chem Eur J. 2003;9:5627–5635. 10.1002/(ISSN)1521-3765 14639646

[7] Meiser F, Cortez C, Caruso F, et al Biofunctionalization of fluorescent rare-earth-doped Lanthanum Phosphate Colloidal Nanoparticles. Angew Chem Int Ed. 2004;43:5954–5957. 10.1002/(ISSN)1521-3773 15547904

[8] Lehmann O, Meyssamy H, Kompe K, et al Synthesis, growth, and Er^3+^ luminescence of lanthanide phosphate Nanoparticles. J Phys Chem B. 2003;107:7449–7453. 10.1021/jp030012h

[9] Wang M, Li M, Yang MY, et al NIR-induced highly sensitive detection of latent fingermarks by NaYF4:Yb, Er upconversion Nanoparticles in a dry powder state. Nano Res. 2015;8:1800–1810. 10.1007/s12274-014-0686-6 27818741 PMC5091657

[10] Yang J, Li CX, Cheng ZY, et al Size-tailored synthesis and luminescent properties of one-dimensional Gd_2_O_3_: Eu^3+^ nanorods and microrods. J Phys Chem C. 2007;111:18148–18154. 10.1021/jp0767112

[11] Mao CB, Li HD, Cui FZ, et al The functionalization of titanium with EDTA to induce biomimetic mineralization of hydroxyapatite. J Mater Chem. 1999;9:2573–2582. 10.1039/a901309a

[12] Huang P, Chen DQ, Wang YS Host-sensitized multicolor tunable luminescence of lanthanide ion doped one-dimensional YVO_4_ nano-crystals. J Alloy Compd. 2011;509:3375–3381. 10.1016/j.jallcom.2010.12.069

[13] Fang YP, Xu AW, Song RQ, et al Systematic synthesis and characterization of single-crystal Lanthanide Orthophosphate nanowires. J Am Chem Soc. 2003;25:16025–16304. 10.1021/ja037280d 14677994

[14] Schuetz P, Caruso F Electrostatically assembled fluorescent thin films of rare-earth-doped Lanthanum Phosphate Nanoparticles. Chem Mater. 2002;14:4509–4516. 10.1021/cm0212257

[15] Li J-G, Li XD, Sun XD, et al Monodispersed colloidal spheres for uniform Y_2_O_3_: Eu^3+^ red-phosphor particles and greatly enhanced luminescence by simultaneous Gd^3+^ doping. J Phys Chem C. 2008;112:11707–11716. 10.1021/jp802383a

[16] Li JK, Li J-G, Liu SH, et al Greatly enhanced Dy^3+^ emission via efficient energy transfer in gadolinium aluminate garnet (Gd_3_Al_5_O_12_) stabilized with Lu^3+^ . J Mater Chem C. 2013;1:7614–7622. 10.1039/c3tc31413h

[17] Zhang LH, Yin ML, You HP, et al Mutifuntional GdPO_4_:Eu^3+^ hollow spheres: synthesis and magnetic and luminescent properties. Inorg Chem. 2011;50:10608–10613. 10.1021/ic200867a 21970439

[18] Huang C-C, Lo Y-W, Kuo W-S, et al Facile preparation of self-assembled hydrogel-like GdPO_4_·H_2_O nanorods. Langmuir. 2008;24:8309–8313. 10.1021/la800847d 18570444

[19] Yu LX, Li DC, Yue MX, et al Dependence of morphology and photoluminescent properties of GdPO_4_: Eu^3+^ nanostructures on synthesis condition. Chem Phys. 2006;326:478–482. 10.1016/j.chemphys.2006.03.008

[20] Guo H, Li F, Wei RF, et al Elaboration and luminescent properties of Eu/Tb co-doped GdPO4-Based glass ceramics for white LEDs. J Am Ceram Soc. 2012;95:1178–1181. 10.1111/j.1551-2916.2012.05097.x

[21] Wu XL, Li J-G, Zhu Q, et al One-step freezing temperature crystallization of layered rare-earth hydroxide (Ln_2_(OH)_5_NO_3_·nH_2_O) nanosheets for a wide spectrum of Ln (Ln = Pr-Er, and Y), anion exchange with fluorine and sulfate, and microscopic coordination probed via photoluminescence. J Mater Chem C. 2015;3:3428–3437. 10.1039/C4TC02681K

[22] Gandara F, Perles J, Snejko N, et al Layered rare-earth hydroxides: a class of pillared crystalline compounds for intercalation chemistry. Angew Chem Int Ed. 2006;45:7998–8001. 10.1002/(ISSN)1521-3773 17096440

[23] Geng FX, Matsushita Y, Ma RZ, et al General synthesis and structural evolution of a layered family of Ln(8)(OH)(20)Cl-4·nH(2)O (Ln = Nd, Sm, Eu, Gd, Tb, Dy, Ho, Er, Tm, and Y). J Am Chem Soc. 2008;130:16344–16350. 10.1021/ja807050e 18998680

[24] Zhu Q, Li J-G, Zhi CY, et al Layered rare-earth hydroxides (LRHs) of (Y_1-x_Eu_x_)_2_(OH)_5_NO_3_·nH_2_O (x = 0-1): structural variations by Eu^3+^ doping, phase conversion to oxides, and the correlation of photoluminescence behaviors. Chem Mater. 2010;22:4204–4213. 10.1021/cm1011586

[25] Lu B, Li J-G, Sun XD, et al Effects of Gd substitution on sintering and optical properties of highly transparent (Y_0.95-x_Gd_x_Eu_0.05_)(2)O(3) Ceramics. J Am Ceram Soc. 2015;98:2480–2487. 10.1111/jace.2015.98.issue-8

[26] Wang ZH, Li J-G, Zhu Q, et al Sacrificial conversion of layered rare-earth hydroxide (LRH) nanosheets into (Y_1−x_Eu_x_)PO_4_ nanophosphors and investigation of photoluminescence. Dalton Trans. 2016;45:5290–5299. 10.1039/C5DT01983D 26898332

[27] Yang M, You HP, Jia G, et al Selective synthesis of hexagonal and monoclinic LaPO_4_:Eu^3+^ nanorods by a hydrothermal method. J Cryst Growth. 2009;311:4753–4758. 10.1016/j.jcrysgro.2009.09.027

[28] Wang ZH, Li J-G, Zhu Q, et al. Hydrothermal conversion of layered hydroxide nanosheets into (Y_0.95_Eu_0.05_)PO_4_ and (Y_0.96-x_Tb_0.04_Eu_x_)PO_4_ (x = 0-0.10) nanocrystals for red and color-tailorable emission. RSC Adv. 2016;6:22690–22699. 10.1039/C6RA00434B

[29] Yan RX, Sun XM, Wang X, et al Crystal structures, anisotropic growth, and optical properties: controlled synthesis of Lanthanide Orthophosphate one-dimensional nanomaterials. Chem Eur J. 2005;11:2183–2195. 10.1002/(ISSN)1521-3765 15714538

[30] Firsching FH, Brune SN Solubility products of the trivalent rare-earth phosphates. J Chem Eng Data. 1991;36:93–95. 10.1021/je00001a028

[31] Deng H, Liu CM, Yang SH, et al Additive-Mediated splitting of Lanthanide Orthovanadate Nanocrystals in water: morphological evolution from rods to sheaves and to spherulites. Cryst Growth Des. 2008;8:4432–4439. 10.1021/cg800207z

[32] Momma K, Izumi F VESTA: a three-dimensional visualization system for electronic and structural analysis. J Appl Crystallogr. 2008;41:653–658. 10.1107/S0021889808012016

[33] Geng DL, Shang MM, Yang DM, et al Tunable luminescence and energy transfer properties in KCaGd(PO4)(2):Ln(3+)/Mn(2+) (Ln = Tb, Dy, Eu, Tm; Ce, Tb/Dy) phosphors with high quantum efficiencies. J Mater Chem. 2012;22:23789–23798. 10.1039/c2jm34991d

[34] Hezel A, Ross SD Forbidden transitions in infra-red spectra of teterhedral anions 3. spectra-structure correlations in perchlorates sulphates and phosphates of formular MXO_4_ . Spectrochim Acta. 1996;22:1949–1961.

[35] Murphy KE, Altman MB, Wunderlich B Monoclinic-to-trigonal transformation in selenium. J Appl Phys. 1977;48:4122–4131. 10.1063/1.323439

[36] Kijkowska R, Cholewka E, Duszak B X-ray diffraction and Ir-absorption characteristics of lanthanide orthophosphates obtained by crystallisation from phosphoric acid solution. J Mater Sci. 2003;38:223–228. 10.1023/A:1021188810349

[37] Li YH, Hong GY Synthesis and luminescence properties of nanocrystalline Gd_2_O_3_: Eu^3+^ by combustion process. J Lumin. 2007;124:297–301. 10.1016/j.jlumin.2006.03.016

[38] Judd BR Optical absorption intensities of rare-earth ions. Phys Rev. 1962;127:750–761. 10.1103/PhysRev.127.750

[39] Gorller-Walrand C, Binnemans K Handbook on the physics and chemistry of rare earths. Amsterdam (Netherlands): Elsevier Academic Press; 1998 Chapter 167, Spectral intensities of f-f transitions; p. 101–264.

[40] Sá Ferreiraa RA, Nobrea SS, Granadeirob CM, et al A theoretical interpretation of the abnormal ^5^D_0_-^7^F_4_ intensity based on the Eu^3+^ local coordination in the Na_9_[EuW_10_O_36_]·14H_2_O polyoxometalate. J Lumin. 2006;121:561–567. 10.1016/j.jlumin.2005.12.044

[41] Lu SZ, Zhang JH, Zhang JS, et al Remarkably enhanced photoluminescence of hexagonal GdPO4·nH2O: Eu with decreasing size. Nanotechnol. 2010;21:365709 10.1088/0957-4484/21/36/365709 20705974

[42] Yaiphaba N, Ningthoujam RS, Singh NS, et al Luminescence, lifetime, and quantum yield studies of redispersible Eu^3+^-doped GdPO_4_ crystalline nanoneedles: Core-shell and concentration effects. J Appl Phys. 2010;107:034301 10.1063/1.3294964

[43] Zhu Q, Li J-G, Ma RZ, et al Well-defined crystallites autoclaved from the nitrate/NH4OH reaction system as the precursor for (Y, Eu)2O3 red phosphor: Crystallization mechanism, phase and morphology control, and luminescent property. J Solid State Chem. 2012;192:229–237. 10.1016/j.jssc.2012.04.015

