# Supplementary material for: EDTA-assisted phase conversion synthesis of (Gd0.95RE0.05)PO4 nanowires (RE = Eu, Tb) and investigation of photoluminescence
Source: Sci Technol Adv Mater. 2017 Jun 28;18(1):447–57. doi: 10.1080/14686996.2017.1338495 (PMC5507148; doi:10.1080/14686996.2017.1338495)
Supplement: Supplementary_information.doc [file tsta_a_1338495_sm7153.doc]

**Supplementary Information**

**EDTA-assisted phase conversion synthesis of (Gd_0.95_RE_0.05_)PO_4_ nanowires (RE = Eu, Tb) and investigation of photoluminescence**

Zhihao Wang^a,b,c^, Ji-Guang Li^a,b,c*^, Qi Zhu^a,b^, Zhengrong Ai^a^, Xiaodong Li^a,b^, Xudong Sun^a,b,d^, Byung-Nam Kim^c^, Yoshio Sakka^c^

^a^*Key Laboratory for Anisotropy and Texture of Materials, Northeastern University, Shenyang, Liaoning 110819, China*

^b^*Institute for Ceramics and Powder Metallurgy, School of Materials Science and Engineering, Northeastern University, Shenyang, Liaoning 110819, China*

^c^*Research Center for Functional Materials, National Institute for Materials Science, Namiki 1-1, Tsukuba, Ibaraki 305-0044, Japan*

^d^*School of Environmental and Chemical Engineering, Dalian University, Dalian, Liaoning 116622, China*

*Corresponding author

Dr. Ji-Guang Li

National Institute for Materials Science

Tel: +81-29-860-4394

E-mail: [li.jiguang@nims.go.jp](mailto:li.jiguang@nims.go.jp)


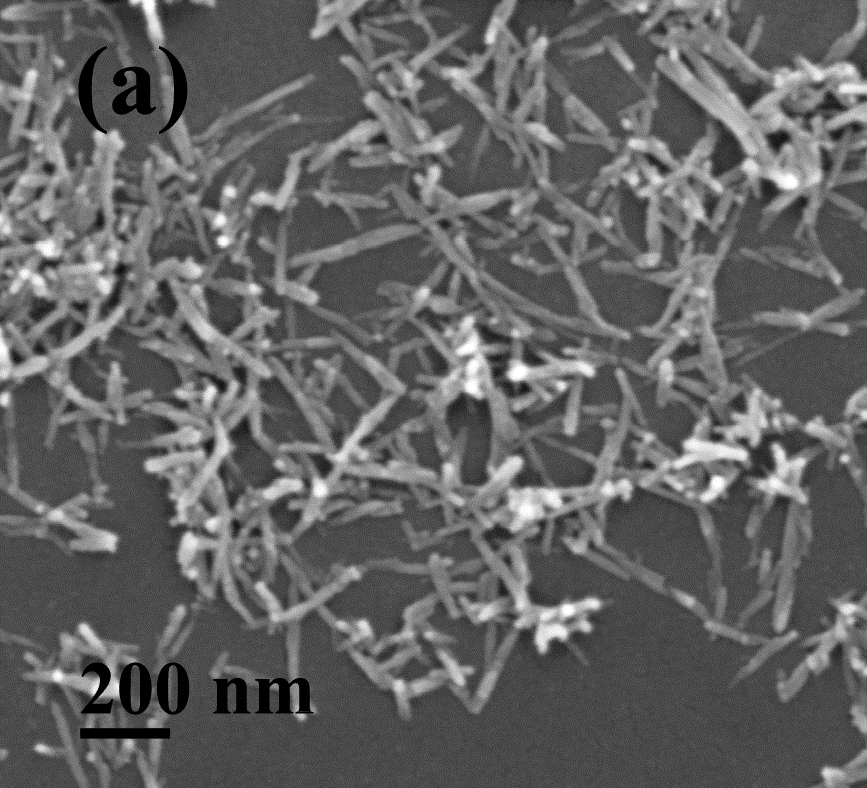

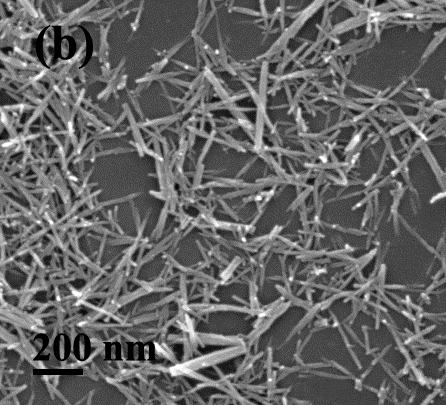

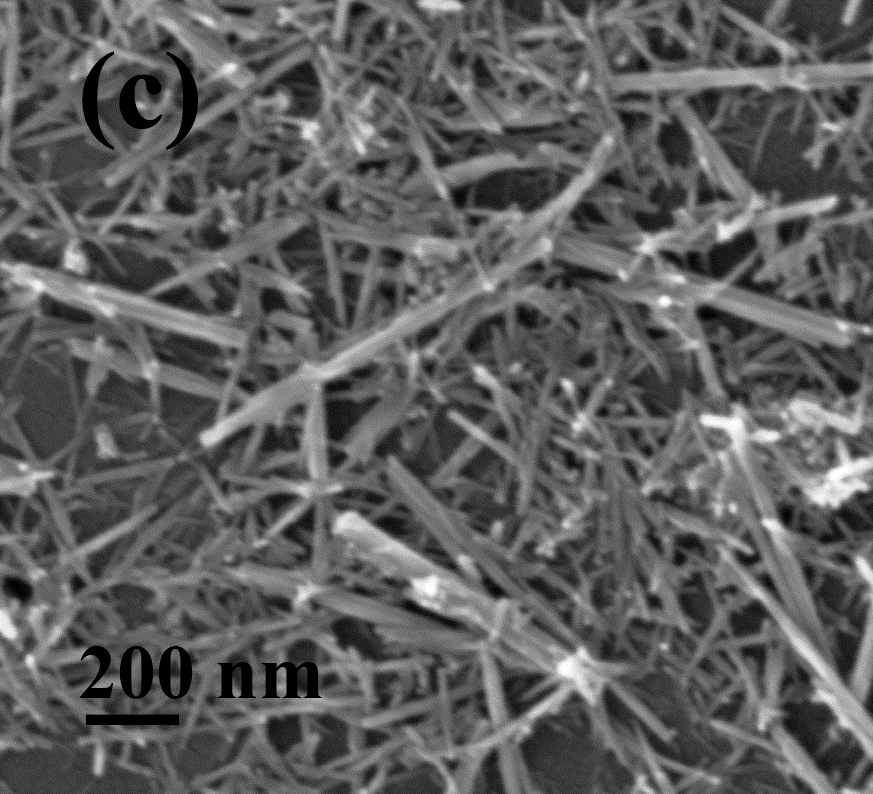


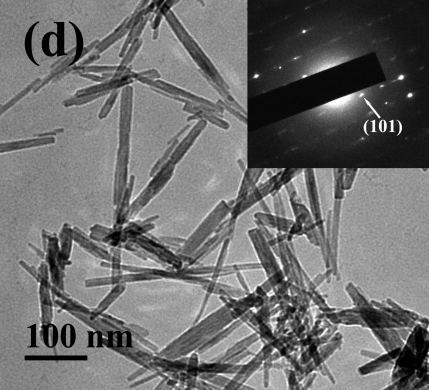

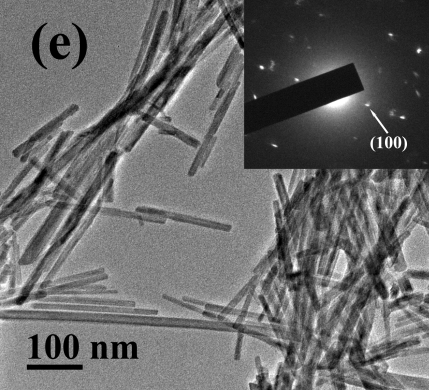


**Figure S1.** FE-SEM (a-c) and TEM (d, e) micrographs showing morphologies of the products synthesized with the different hydrothermal temperatures of (a, d) 120 ^o^C (S10), (b, e) 180 ^o^C (S11) and (c) 200 ^o^C (S12). The insets in (d, e) are the corresponding SAED patterns.

**Figure S2.** TG profile of the 150 ^o^C hydrothermal product.


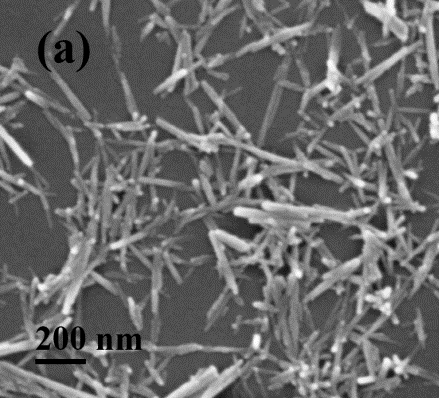

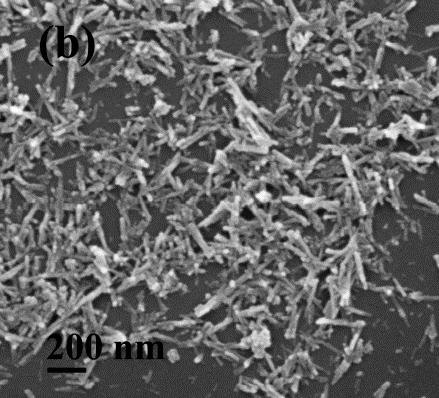

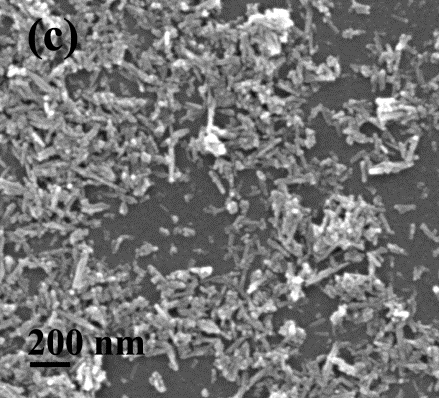


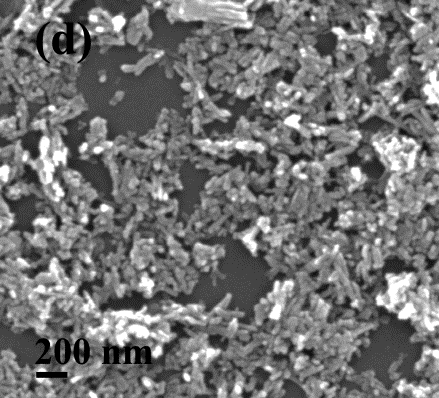

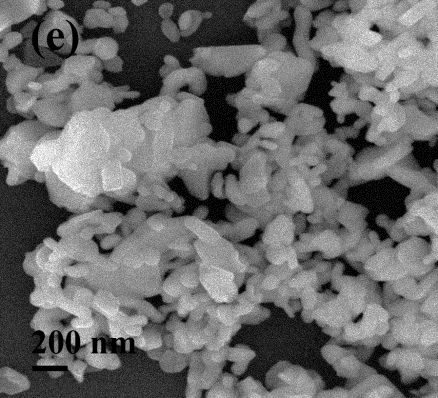

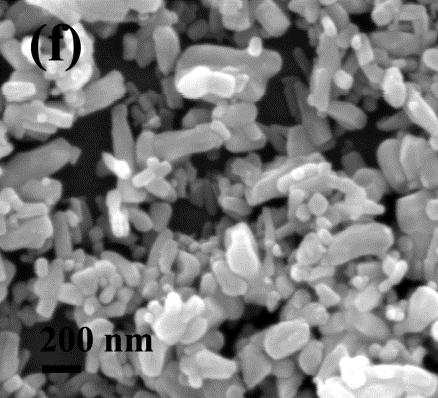


**Figure S3.** FE-SEM micrographs showing typical morphologies of the products calcined from sample S5 at (a) 500, (b) 600, (c) 900, (d) 1000, (e) 1100, and (f) 1200 ^o^C.

**Figure S4.** XRD patterns for the (Gd_0.95_Tb_0.05_)PO_4_·*n*H_2_O sample synthesized under the same hydrothermal conditions of S5 (a) and the product calcined at 500 ^o^C (b).


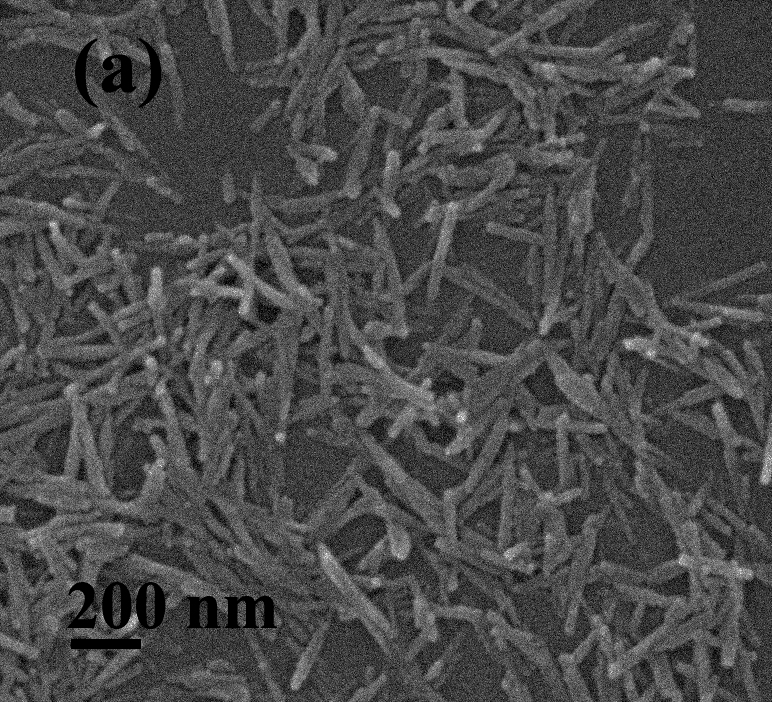

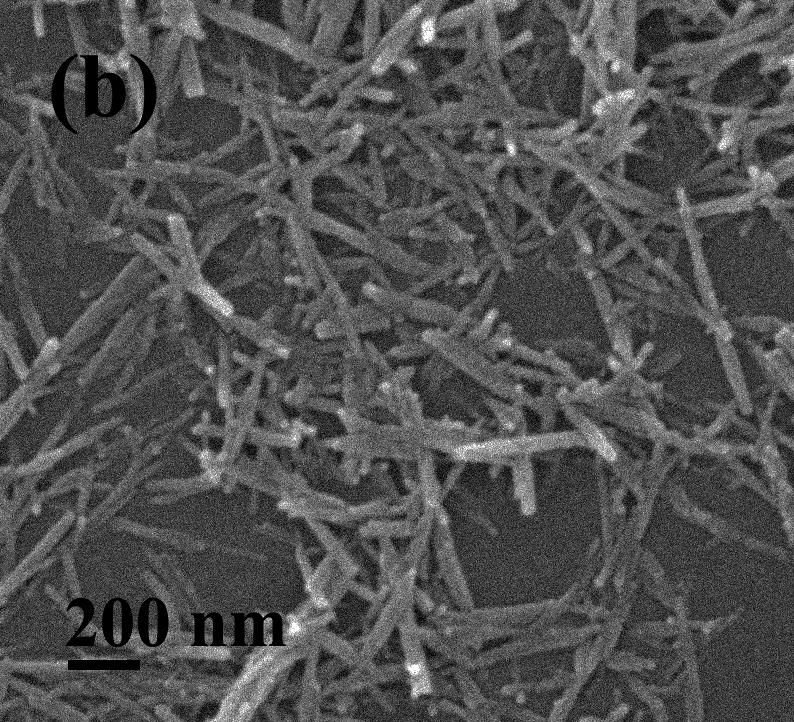


**Figure S5.** SEM morphologies for the (Gd_0.95_Tb_0.05_)PO_4_·*n*H_2_O sample synthesized under the same hydrothermal conditions of S5 (a) and the product calcined at 500 ^o^C (b).

**Figure S6.** PL spectrum of the (Gd_0.95_Eu_0.05_)PO_4_ phosphor (calcined at 1000 ^o^C) obtained by exciting Gd^3+^ at 272 nm (a) and schematic show of the energy transfer from Gd^3+^ to Eu^3+^ (b).

**Figure S7.** PL spectrum of the (Gd_0.95_Tb_0.05_)PO_4_ phosphor (calcined at 500 ^o^C) obtained by exciting Gd^3+^ at 272 nm (a) and schematic show of the energy transfer from Gd^3+^ to Tb^3+^ (b).

**Figure S8.** Fluorescence decay kinetics for the 593 nm red emission of Eu^3+^ (A, red) and the 546 nm green emission of Tb^3+^ (B, green). The results of exponential fitting are presented in A and B with black lines. Parts (a)-(f) in panel A are for sample S5 (a) and the products calcined from S5 at (b) 500, (c) 900, (d) 1000, (e) 1100 and (f) 1200 ^o^C. Parts (a) and (b) in panel B are for (Gd_0.95_Tb_0.05_)PO_4_·*n*H_2_O (a) and the product calcined at 500 ^o^C (b).

**Figure S9.** ^5^D_0_→^7^F_1_/^5^D_0_→^7^F_2_ and ^5^D_0_→^7^F_1_/^5^D_0_→^7^F_4_ intensity ratios as a function of the calcination temperature.
